# Advanced Glycation End-Products and Hyperglycemia Increase Angiopoietin-2 Production by Impairing Angiopoietin-1-Tie-2 System

**DOI:** 10.1155/2019/6198495

**Published:** 2019-11-11

**Authors:** Alessandra Puddu, Roberta Sanguineti, Davide Maggi, Massimo Nicolò, Carlo E. Traverso, Renzo Cordera, Giorgio L. Viviani

**Affiliations:** ^1^Department of Internal Medicine and Medical Specialties, University of Genova, Genova 16132, Italy; ^2^Department of Neuroscience, Ophthalmology and Genetics, University of Genova, Genova 16132, Italy; ^3^Fondazione per la Macula Onlus, Genova 16132, Italy

## Abstract

The angiopoietin-Tie-2 system plays a crucial role in the maintenance of endothelial integrity. Hyperglycemia and advanced glycation end-products (AGEs) are involved in endothelial cell dysfunction responsible of the pathogenesis of microvascular complications of diabetes. Here, we investigated whether glycated serum (GS) or hyperglycemia (HG) affect the angiopoietin-Tie-2 system in the microvascular endothelial cells HMEC-1. We found that culture for 5 days in the presence of AGEs and HG (alone or in combination) decreased cell proliferation, increased reactive oxygen species (ROS) production, and reduced ratio between the oxidized and the reduced form of glutathione. Since angiopoietin-1 (Ang-1) signaling regulates angiopoietin-2 (Ang-2) expression through inactivation of the forkhead transcription factor FoxO1, we investigated intracellular signaling of Ang-1 and expression of Ang-2. HG and AGEs reduced phosphorylation of Akt and abrogated phosphorylation of FoxO1 induced by Ang-1 without affecting neither Tie-2 expression nor its activation. Furthermore, AGEs and/or HG induced nuclear translocation of FoxO1 and increased Ang-2 production. In conclusion, we demonstrated that both hyperglycemia and AGEs affect the angiopoietin-Tie-2 system by impairing Ang-1/Tie-2 signaling and by increasing Ang-2 expression. These results suggest that therapeutic strategies useful in preventing or delaying the onset of diabetic vascular complications should be aimed to preserve Ang-1 signaling.

## 1. Introduction

The angiopoietin-Tie-2 system plays a crucial role in vessel maturation and quiescence and modulates the maintenance of endothelial integrity [1]. The angiopoietin growth factor-1 (Ang-1), which is produced by perivascular cells, is an endothelial-specific protective factor and contributes to vessel integrity by activating the tyrosine kinase receptor Tie-2 expressed by endothelial cells [2]. Binding of Ang-1 to Tie-2 leads to different intracellular signals mainly mediated by the phosphatidylinositol 3-kinase (PI3K)/Akt pathway [3], which contribute to the maintenance of the resting phenotype and regulate survival, migration, and permeability of endothelial cells [4]. Conversely, the angiopoietin growth factor-2 (Ang-2), which is produced by the endothelial cells, acts as a dominant negative ligand of Tie-2, thereby leading to vessel-destabilization and favoring the proangiogenic and inflammatory response to growth factors and cytokines [5, 6]. Once produced, Ang-2 is stored in Weibel-Palade bodies and is released in response to inflammatory stimuli [4]. Interestingly, expression of Ang-2 is regulated by Akt signaling activated by Ang-1 through phosphorylation and inactivation of the forkhead transcription factor FoxO1 [7]. In turn, FoxO1 targets Ang-2, leading to a negative-feedback loop that results in reduced Ang-2 gene expression [7, 8].

Type 2 diabetes mellitus, a metabolic disease characterized by chronic hyperglycemia and low-grade inflammation, lead to several vascular complications [9]. It is well known that chronic hyperglycemia leads to accelerate formation of advanced glycation end-products (AGEs), a heterogeneous group of compounds resulted from the nonenzymatic reaction of reducing sugars with free amino group of proteins [10]. AGEs may exert adverse effects through several mechanisms, including the formation of the protein cross-link that alters the structure and function of the extracellular matrix, the production of reactive oxygen species (ROS), and the interaction with specific receptors [11–13]. Furthermore, AGEs are responsible for the “metabolic memory” [14]. The detrimental effects of hyperglycemia and AGEs have an important role in the development and the severity of diabetic complications, also due to the impairment of antioxidant defenses, such as glutathione [15–18].

Endothelial dysfunction, including defect in angiogenesis, increased endothelial permeability, elevated leukocyte adhesion, and impaired nitric oxide action, is implicated in vascular complications of diabetes [19, 20]. Recent findings suggest that hyperglycemia may predispose to endothelial dysfunction by affecting the angiopoietin-Tie-2 system [21]. The aim of this study was to investigate the effects of hyperglycemia and AGEs in regulating the angiopoietin-Tie-2 system in endothelial cells and to identify the possible mechanisms responsible for this process.

## 2. Materials and Methods

### 2.1. Preparation of AGEs

Glycated serum (GS) was prepared by adding 50 mmol/L ribose to heat-inactivated (56°C for one hour) FBS, as described previously [22]. Aliquots of FBS were processed the same way but without ribose (nonglycated serum (NGS)) and used for standard medium preparation. Pentosidine content was evaluated as a measure of protein glycation, as previously described [19]. The concentration of pentosidine in the experimental media containing NGS was 70 pmol/mL, whereas the concentration of pentosidine in the experimental media containing GS was 400 pmol/mL which corresponds to the levels within the pathophysiological range detected in the plasma of diabetic patients.

### 2.2. Cell Culture and Experimental Conditions

HMEC-1 cells derived from human dermal microvascular endothelium were purchased from ATCC (Manassas, VA). Cells were cultured in MCDB131 medium supplemented with 10 ng/mL epidermal growth factor, 1 *μ*g/mL hydrocortisone, 10 mM glutamine, and 10% FBS. The medium was replaced every 2 days. Cells were grown to confluence, removed with Trypsin-EDTA (Sigma-Aldrich, Milan, Italy), and then seeded in multiwell plates for all experiments. Before each experiment, confluent cells were washed twice with PBS (Cambrex Bio Science) and then cultured in standard medium (CTR), in the presence of glycated serum (AGEs), 25 mM glucose (HG), and with their combination (AGEs+HG).

### 2.3. Cell Viability

To evaluate cell proliferation, HMEC-1 cells were plated in a 96-well plate (2 × 10^4^ cells/well) and cultured for 5 days as described above. Cell proliferation rate was determined using the Cell Titer 96 Aqueous One Solution Cell Proliferation Assay (Promega, Milan, Italy) according to the manufacturer's instructions. Briefly, it is a colorimetric method that determines the number of viable cells via MTS tetrazolium reduction into a colored formazan product directly proportional to the number of living cells in culture [22]. Values were expressed as arbitrary units.

### 2.4. Reactive Oxygen Species Detection

Intracellular reactive oxygen species (ROS) level was measured using the cell-permeable fluorescent probe, 2′,7′-dichlorofluorescein diacetate (DCFH-DA) (Sigma-Aldrich, Milan, Italy). In brief, cells were seeded into 6-well culture plates and treated for 5 days as previously described, then washed twice with Hank's buffered salt solution (HBSS) and incubated with fresh DCFH-DA (25 *μ*M) in HBSS for 30 min at 37°C in 5% CO_2_. After that, cells were washed twice in HBSS, and wells were filled with 1 mL HBSS before fluorescence acquisition in a plate reader (TECAN InfinitePro200) (Ex : *λ*485/Em : *λ*535 nm). Fluorescent emission was normalized to total protein content. Results were expressed as arbitrary units.

### 2.5. Ratio between the Oxidized (GSSG) and the Reduced (GSH) Form of Glutathione

To evaluate the intracellular content of glutathione (GSH), cells were plated in 6-well dishes and cultured as described above. Quantification of glutathione was performed using Cayman's GSH askay Kit according to the manufacturer's instructions as previously reported [23].

### 2.6. Cell Lysis and Ang-1-Tie-2 Signaling

At the end of the experiments, a set of HMEC-1 cells was lysed in RIPA buffer (50 mmol/l Tris HCl pH 7.5, 150 mmol/l NaCl, 1% NP40, and 0.1% SDS), supplemented with protease and phosphatase inhibitors. Another set of HMEC-1 cells was incubated in endothelial cell basal medium lacking FBS or growth supplements. After 2 hours, cells were exposed for 30 min to PBS (control) or Ang-1 (200 ng/ml). Medium was removed and the cells were lysed in RIPA buffer. Cell lysates were assayed for phosphorylated and total levels of Tie-2 receptors, Akt, and FoxO1 using immunoblotting. Protein concentration of each sample was determined using BCA Protein Assay Kit (Pierce Biotechnology, Rockford, IL, USA).

### 2.7. Cell Lysis and Subcellular Fractionation

At the end of each experiments, a set of HMEC-1 cells was lysed in RIPA buffer, supplemented with protease and phosphatase inhibitors. Another set of HMEC-1 cells was processed for subcellular fractionation using the Subcellular Protein Fractionation Kit (Pierce Biotechnology, Rockford, IL, USA) according to the manufacturer's instructions. Briefly, various cellular compartments were isolated by sequential addition of different extraction buffers to the cell pellet. Each subcellular fraction was collected after centrifugation and stored at -80°C. Protein concentration of each sample was determined using BCA Protein Assay Kit (Pierce Biotechnology, Rockford, IL, USA).

### 2.8. Immunoblotting Analysis

Equal protein amounts of total cell lysate, cytosolic, and nuclear fractions were separated on 4-20% SDSPAGE and transferred onto nitrocellulose. Filters were blocked in 5% nonfat dried milk and incubated overnight at 4°C with primary specific antibodies (anti-*β*-actin, anti Akt, anti-phospho-Akt (Ser473), anti-angiopoietin-2, anti-FoxO1, anti-phospho-FoxO1 (Ser256), anti-GAPDH, anti-Histone H3, anti-Tie-2, and anti-phospho-Tie-2 (Tyr992) from Cell Signaling Technology, Beverly, MA, USA). Secondary specific horseradish peroxidase-linked antibodies were added for 1 h at room temperature. Bound antibodies were detected using the enhanced chemiluminescence lighting system (ECL Plus), according to the manufacturer's instructions. Each membrane was stripped (Restore PLUS Western Blot Stripping Buffer, Pierce Biotechnology, Rockford, IL, USA) and probed for *β*-actin to verify equal protein loading. Bands of interest were quantified by densitometry using the Alliance software. Results were expressed as percentages of CTR (defined as 100%).

### 2.9. Statistical Analysis

The results are representative of at least 3 experiments. All analyses were carried out with the GraphPad Prism 4.0 software (GraphPad Software, San Diego, CA, USA). Data were expressed as the mean ± SEM and then analyzed using one-way ANOVA followed by Bonferroni's multiple comparison test. A *p* value of <0.05 was considered statistically significant.

## 3. Results

### 3.1. AGEs and HG Decrease Cell Viability and Increase Oxidative Stress

**Figure 1:**
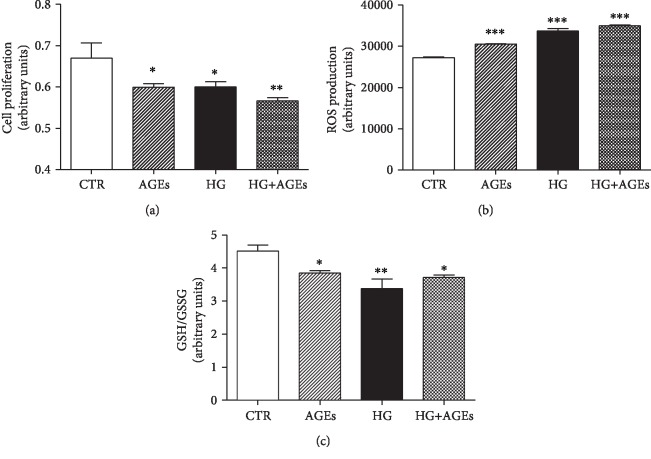
Cell viability and oxidative stress in HMEC-1 cells cultured for 5 days in standard medium (CTR), in the presence of glycated serum (AGEs), 25 mM glucose (HG), and with their combination (AGEs+HG). (a) Cell proliferation rate (*n* = 4, ^∗^*p* < 0.05 and ^∗∗^*p* < 0.01 vs. CTR). (b) Evaluation of intracellular ROS production (*n* = 4, ^∗∗∗^*p* < 0.001 vs. CTR) (c) Ratio between the reduced (GSH) and the oxidized (GSSG) forms of glutathione (*n* = 4, ^∗^*p* < 0.05 and ^∗∗^*p* < 0.01 vs. CTR). Values shown indicate the mean SEM of at least 3 independent experiments.

Firstly, we investigate whether diabetic condition affect viability of endothelial cells, and we found that culture of HMEC-1 cells for 5 days with AGEs, HG, or their combination significantly reduced cell proliferation (Figure 1(a)). Reactive oxygen species (ROS) are reactive intermediates of molecular oxygen that act as important second messengers within the cells. An imbalance between generation of reactive ROS and antioxidant defense systems represents the primary cause of endothelial dysfunction. The incubation of HMEC-1 with AGEs or HG significantly increase ROS intracellular production as compared to cells incubated with control medium (Figure 1(b)).

Glutathione (GSH) is one of the most important and potent antioxidants capable of preventing damage caused by ROS by maintaining redox balance [24]. In the presence of oxidative stress, it is oxidized to the disulphide dimer GSSG. As shown in Figure 1(c), exposure of HMEC-1 cells to AGEs, HG, or their combination induced a reduction of the GSH/GSSG ratio.

### 3.2. Treatment with AGEs and HG Affects Ang-1 Signaling

During physiological process, ROS act as important mediators of intracellular signaling and play an important role in cellular differentiation and maintenance of homeostasis [25]. However, overproduction of ROS contributes to endothelial dysfunction. To verify whether increased ROS production and reduction of the GSH/GSSG ratio is associated with impaired Ang-1 signaling, we investigated expression of Tie-2 and activation of Ang-1 signaling cascade.

**Figure 2:**
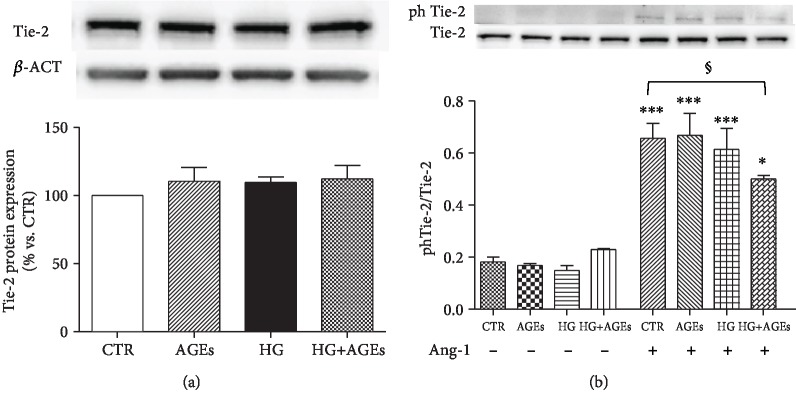
AGEs and HG did not alter the expression and phosphorylation of Tie-2. (a) Western immunoblotting of Tie-2 in HMEC-1 cells cultured for 5 days in CTR, AGEs, HG, and HG+AGEs. (b) After the 5-day treatment described above, HMEC-1 cells were incubated for 2 hours in serum-free medium and, then, exposed for 30 min to PBS (control) or Ang-1 (200 ng/ml). Then, Tie-2 and phosphoTie-2 were analyzed. Representative western blot analysis and quantification of densitometries of western blot band. Data were expressed as mean ± SEM of fold induction relative to *β*-actin (a) or to relative expression of Tie-2 and phTie-2 (b) (*n* = 4). ^∗^*p* < 0.05 and ^∗∗∗^*p* < 0.001 vs. same culture condition in the absence of Ang-1.

Culture of HMEC-1 cells with AGEs, HG, or their combination did not affect expression of Tie-2 (Figure 2(a)).

When HMEC-1 cells are cultured under standard condition, angiopoietin-1 induces an about threefold increment of Tie-2 phosphorylation in comparison with unstimulated cells. Culture with AGEs or HG did not affect the ability of angiopoietin-1 to induce phosphorylation of Tie-2; however, the combination of AGEs and HG significantly reduced Ang-1 induced Tie-2 phosphorylation (Figure 2(b)).

**Figure 3:**
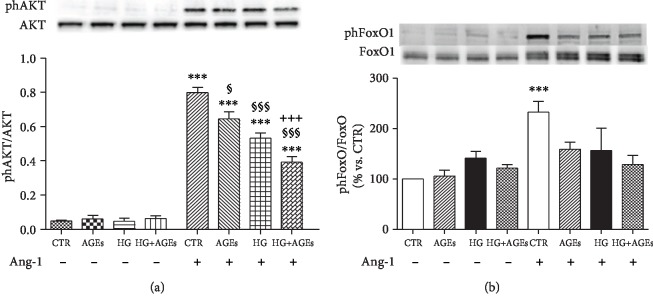
AGEs and HG reduced phosphorylation of AKT and FoxO1 induced by Ang-1. After the 5-day treatment described above, HMEC-1 cells were incubated for 2 hours in serum-free medium and, then exposed for 30 min to PBS (control) or Ang-1 (200 ng/ml). Then, phAKT and AKT (a) and phFoxO1 and FoxO1 (b) were analyzed. Representative western blot analysis and quantification of densitometries of western blot band. Data were expressed as mean ± SEM of fold induction relative to unphosphorylated protein (*n* = 4). ^∗∗∗^*p* < 0.001 vs. the same culture condition in the absence of Ang-1; ^$^*p* < 0.5 and ^$$$^*p* < 0.001 vs. CTR+Ang-1; and ^+++^*p* < 0.001 HG+AGEs+Ang-1 vs. HG+Ang-1.

Akt is considered a major angiogenic downstream mediator of the Ang-1-Tie-2 signaling pathway [3]. Stimulation of HMEC-1 cells with Ang-1 induced a strong increase of Akt phosphorylation. Treatment with AGEs or HG reduced phosphorylation of Akt induced by Ang-1; combination of AGEs and HG further impaired the ability of Ang-1 to phosphorylate Akt (Figure 3(a)).

Ang-1-induced Akt activation is a potent inhibitor of FoxO1 [7]. Therefore, we investigated FoxO1 phosphorylation under Ang-1 stimulation. In control condition, phosphorylation of FoxO1 increased of about 2.5-fold when cells are stimulated with Ang-1. On the contrary, we did not observe any significant increase of FoxO1 phosphorylation when cells are cultured in diabetic milieu (Figure 3(b)).

**Figure 4:**
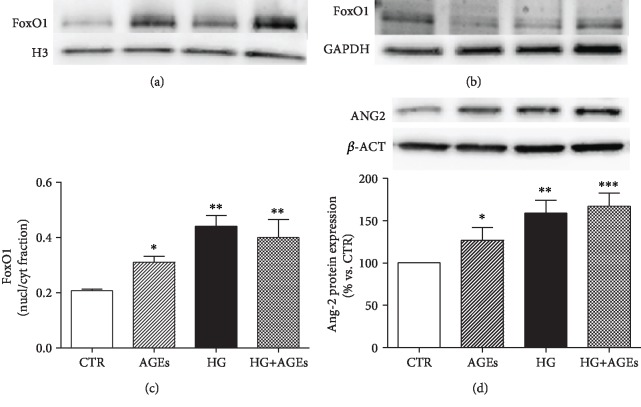
AGEs and HG increased expression of Ang-2. HMEC-1 cells were cultured for 5 days in standard medium (CTR), in the presence of glycated serum (AGEs), 25 mM glucose (HG), and with their combination (AGEs+HG). Western blot analysis of nuclear (a) and cytosolic (b) localization of FoxO1. Histone H3 and GAPDH amounts were analyzed as loading controls. (c) Graph represents relative ratio between nuclear and cytosolic fractions (*n* = 3, ^∗^*p* < 0.05 and ^∗∗^*p* < 0.01 vs. CTR). (d) Western immunoblotting of Ang-2. Representative western blot analysis and quantification of densitometries of western blot band. Data were expressed as mean ± SEM of fold induction relative to *β*-actin. Values shown indicate the mean SEM of at least 3 independent experiments (*n* = 3, ^∗^*p* < 0.5, ^∗∗^*p* < 0.01 and _∗∗∗_*p* < 0.001 vs. CTR).

Phosphorylation of FoxO1 leads to nuclear exclusion [26]. To verify whether inability of Ang-1 to induce FoxO1 phosphorylation affects its cellular localization, we investigate subcellular fractions of HMEC-1 cells. Here, we found that HG and AGEs reduced localization of FoxO1 in the cytosolic fraction and induced its translocation into the nucleus (Figures 4(a) and 4(b)), leading to increased ratio between the nuclear and the cytosolic fraction (Figure 4(c)).

FoxO1 is a positive regulator of Ang-2 transcription [8]; thus, we analyzed the expression of Ang-2. Intracellular content of Ang-2 is significantly increased by both AGEs and HG (Figure 4(d)).

## 4. Discussion

In this study, we show that hyperglycemia and AGEs impair the intracellular signaling cascade induced by Ang-1 through activation of FoxO1 thus leading to increased Ang-2 production in the microvascular endothelial cell line HMEC-1. Although AGEs and HG have additive detrimental effects, no significant difference has been found between effects of AGEs and those of HG, confirming that AGEs are deleterious as much as hyperglycemia.

It is well known that generation of oxidative stress by hyperglycemia and AGEs is responsible of endothelial cell dysfunction, cause of microvascular complications of diabetes [27]. Another important role in the etiology of diabetic complications is played by glutathione deficiency [28]. Here, we found that exposure to diabetic milieu increased oxidative stress and altered the redox balance by increasing the ratio between the oxidized and the reduced form of glutathione. This condition may reduce the possibility to counteract the rise in ROS production caused by hyperglycemia and AGEs, further worsening endothelial function and impairing cell proliferation.

Alteration of the angiopoietin-Tie-2 system has a key role in the development of microvascular complication of diabetes, mainly because Ang-1 is a potent vascular protective factor important in maintaining normal endothelial function [29]. It has been reported that incubation of microvascular endothelial cells with 30 mM glucose affects the ability of Ang-1 to activate Tie-2 receptor phosphorylation and inhibits Ang-1-dependent Akt phosphorylation without any decrease in Tie-2 expression [21]. A significant impairment of Ang-1-induced Akt phosphorylation has been also found coupled with downregulation of Tie-2 expression [30]. According to the results of Singh et al. [21], we found that hyperglycemia and AGEs impair the intracellular signaling cascade induced by Ang-1 without changing in Tie-2 expression. However, in our model, this occurs without affecting the ability of Ang-1 to induce phosphorylation of Tie-2, suggesting that diabetic condition impairs also the downstream signal transducers of Tie-2. Akt, which is considered the major mediator of the Tie-2 intracellular signaling pathway, regulates function of endothelial cells, and promotes their survival [3]. Interestingly, our data showed that phosphorylation of Akt is significantly reduced by incubation with HG or AGEs, despite expression and activation of Tie-2 by Ang-1 are unchanged. DeBusk et al. demonstrated that Akt is required and sufficient to mediate Ang-1-endothelial cell survival [3]. Therefore, the reduced ability of Ang-1 to induce Akt phosphorylation, in cells cultured with HG or AGEs, may contribute, together with oxidative stress, to decrease EC viability and function. Akt signaling plays also a key role in regulating Ang-2 expression by deactivating the forkhead transcription factor, FoxO1 [7]. Activity of FoxO1 is regulated by posttranslational modifications, in particular, phosphorylation deactivates FoxO1 determining its cytoplasmic localization [26]. In this study, we found that HG and AGEs prevent phosphorylation of FoxO1 induced by Ang-1, leading, at the same time, to increased translocation of FoxO1 into the nucleus. As expected, increased localization of FoxO1 in the nucleus is associated to a significant increment in production of Ang-2, suggesting that HG and AGEs affect Ang-2 production in endothelial cells by activating FoxO1. Reduced phosphorylation of FoxO1 by AGEs has been observed also in pancreatic beta-cells [31], suggesting the existence of a common mechanism through which AGEs regulate gene transcription.

Once produced, Ang-2 is stored in Weibel-Palade bodies and is released in response to inflammatory stimuli [4]. Clinical studies revealed that the circulating levels of Ang-2 level was increased in patients with diabetes [32, 33], and correlate with vascular complications [29, 34]. The rise in Ang-2 may contribute to destabilize the vasculature, leading to uncontrolled neovascularization, as occurs in the late stage of diabetic retinopathy, or defective angiogenesis, thus impairing wound healing [29]. On the contrary, lower levels of Ang-1 have been found in patients with diabetes [35]. Therefore, the increased Ang-2/Ang-1 ratio may contribute to negatively affect Ang-1-Tie-2 signaling. Our results suggest that HG and AGEs may induce a vicious circle in which the impaired Ang-1 signaling leads to increased Ang-2 expression that, in turn, may contribute to further worsening Ang-1 signaling.

## 5. Conclusions

In this study, we demonstrated that both hyperglycemia and AGEs impair the angiopoietin-Tie-2 system by disrupting Ang-1-Tie-2 signaling and by increasing Ang-2 production via FoxO1 activation. Since Ang-2 can be rapidly released after secretagogue stimulus, increased Ang-2 storage may enhance the responsiveness of endothelial cells to inflammatory or angiogenic cytokines. These results suggest that therapeutic strategies useful in preventing or delaying the onset of diabetic vascular complications should be aimed to prevent formation of AGEs and to preserve Ang-1 signaling in order to maintain tissue homeostasis.

## Data Availability

The data used to support the findings of this study are included within the article.
